# Supplementary material for: Identification of photoperiod-induced specific miRNAs in the adrenal glands of Sunite sheep (Ovis aries)
Source: Front Vet Sci. 2022 Jul 22;9:888207. doi: 10.3389/fvets.2022.888207 (PMC9354845; doi:10.3389/fvets.2022.888207)
Supplement: Supplementary file 1 [file Data_Sheet_1.ZIP › Supplementary file/miRDeep2 file.zip!/miRDeep2 file/Know_Structure/SP42A3/oar-mir-191.pdf]

miRBase precursor : oar-mir-191
Total read count : 99500
oar-miR-191 read count : 99242
remaining reads : 258

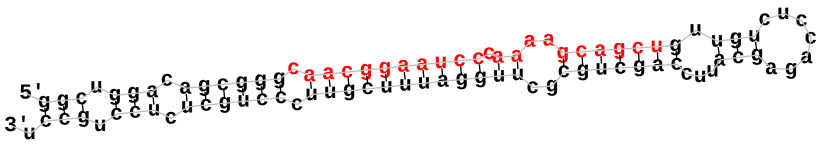

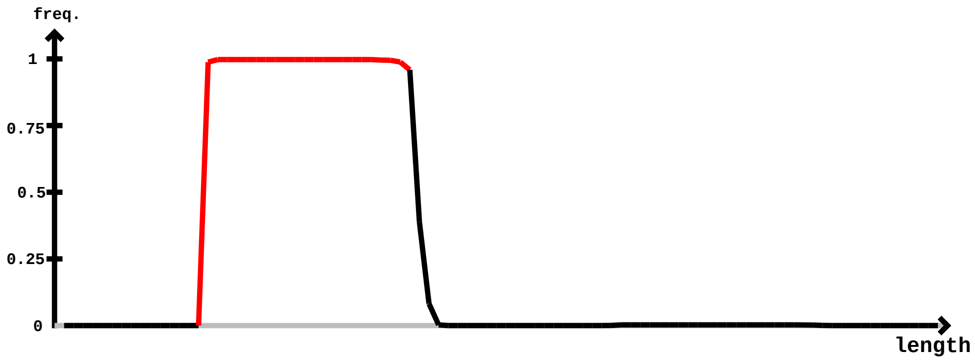


```
                 oar-miR-191
5'- ggcuggacagcgggcaacggaaucccaaaagcagcuguugucuccagagcauuccagcugcgcuuggauuucguucccugcucuccugccu   -3'    exp
    (((.(((.((((((.((((((((((.((..(((((((.(((.......)))...)))))))..))))))))))))).)))))).))).))).    reads   mm   sample
    ..............caacggaaucccaaaagc...........................................................    195     0    seq
    ..............caacggaaucccaaaagca..........................................................    76      0    seq
    ..............caacggaaucccaaaagcag.........................................................    646     0    seq
    ..............caacggaaucccaaaagcagc........................................................    2895    0    seq
    ..............caacggaaucccaaaagcagcu.......................................................    56447   0    seq
    ..............caacggaaucccaaaagcagcug......................................................    30160   0    seq
    ..............caacggaaucccaaaagcagcugu.....................................................    7618    0    seq
    ..............caacggaaucccaaaagcagcuguu....................................................    243     0    seq
    ...............aacggaaucccaaaagc...........................................................    1       0    seq
    ...............aacggaaucccaaaagcag.........................................................    3       0    seq
    ...............aacggaaucccaaaagcagc........................................................    16      0    seq
    ...............aacggaaucccaaaagcagcu.......................................................    261     0    seq
    ...............aacggaaucccaaaagcagcug......................................................    381     0    seq
    ...............aacggaaucccaaaagcagcugu.....................................................    285     0    seq
    ...............aacggaaucccaaaagcagcuguu....................................................    10      0    seq
    ................acggaaucccaaaagcagcu.......................................................    3       0    seq
    ................acggaaucccaaaagcagcug......................................................    1       0    seq
    ................acggaaucccaaaagcagcugu.....................................................    1       0    seq
    .......................................................agcugcgcuuggauuucguuc...............    1       0    seq
    .......................................................agcugcgcuuggauuucguucc..............    1       0    seq
    ........................................................gcugcgcuuggauuucguu................    1       0    seq
    ........................................................gcugcgcuuggauuucguuc...............    11      0    seq
    ........................................................gcugcgcuuggauuucguucc..............    11      0    seq
    ........................................................gcugcgcuuggauuucguuccc.............    12      0    seq
    ........................................................gcugcgcuuggauuucguucccu............    1       0    seq
    ........................................................gcugcgcuuggauuucguucccug...........    1       0    seq
    .........................................................cugcgcuuggauuucguu................    3       0    seq
    .........................................................cugcgcuuggauuucguuc...............    17      0    seq
    .........................................................cugcgcuuggauuucguucc..............    26      0    seq
    .........................................................cugcgcuuggauuucguuccc.............    123     0    seq
    .........................................................cugcgcuuggauuucguucccu............    47      0    seq
    .........................................................cugcgcuuggauuucguucccug...........    1       0    seq
    ..........................................................ugcgcuuggauuucguucccu............    2       0    seq
```
